# Automated extraction of *Camellia oleifera* crown using unmanned aerial vehicle visible images and the ResU-Net deep learning model

**DOI:** 10.3389/fpls.2022.958940

**Published:** 2022-08-11

**Authors:** Yu Ji, Enping Yan, Xianming Yin, Yabin Song, Wei Wei, Dengkui Mo

**Affiliations:** ^1^Key Laboratory of Forestry Remote Sensing Based Big Data and Ecological Security for Hunan Province, Changsha, China; ^2^Key Laboratory of State Forestry and Grassland Administration on Forest Resources Management and Monitoring in Southern Area, Changsha, China; ^3^College of Forestry, Central South University of Forestry and Technology, Changsha, China; ^4^Central South Forest Inventory and Planning Institute of State Forestry Administration, Changsha, China; ^5^Forestry Research Institute of Guangxi Zhuang Autonomous Region, Guangxi, China

**Keywords:** UAV imagery, deep learning, image segmentation, tree-crown detection, *Camellia oleifera*

## Abstract

As one of the four most important woody oil-tree in the world, *Camellia oleifera* has significant economic value. Rapid and accurate acquisition of *C. oleifera* tree-crown information is essential for enhancing the effectiveness of *C. oleifera* tree management and accurately predicting fruit yield. This study is the first of its kind to explore training the ResU-Net model with UAV (unmanned aerial vehicle) images containing elevation information for automatically detecting tree crowns and estimating crown width (CW) and crown projection area (CPA) to rapidly extract tree-crown information. A Phantom 4 RTK UAV was utilized to acquire high-resolution images of the research site. Using UAV imagery, the tree crown was manually delineated. ResU-Net model’s training dataset was compiled using six distinct band combinations of UAV imagery containing elevation information [RGB (red, green, and blue), RGB-CHM (canopy height model), RGB-DSM (digital surface model), EXG (excess green index), EXG-CHM, and EXG-DSM]. As a test set, images with UAV-based CW and CPA reference values were used to assess model performance. With the RGB-CHM combination, ResU-Net achieved superior performance. Individual tree-crown detection was remarkably accurate (Precision = 88.73%, Recall = 80.43%, and F1score = 84.68%). The estimated CW (*R*^2^ = 0.9271, RMSE = 0.1282 m, rRMSE = 6.47%) and CPA (*R*^2^ = 0.9498, RMSE = 0.2675 m^2^, rRMSE = 9.39%) values were highly correlated with the UAV-based reference values. The results demonstrate that the input image containing a CHM achieves more accurate crown delineation than an image containing a DSM. The accuracy and efficacy of ResU-Net in extracting *C. oleifera* tree-crown information have great potential for application in non-wood forests precision management.

## Introduction

*Camellia oleifera*, along with oil palm, coconut, and oil olive, is one of the world’s four most important woody oil-tree, and it is China’s top woody oil-tree. *Camellia oleifera* is widely used in cosmetics, medicine, tannin production, bio-feed, sterilization, and other fields, in addition to its primary use in the production of edible camellia oil (Zhang et al., 2013). However, the management of *C. oleifera* continues to rely excessively on manual labor and possesses insufficient scientific and technological support. For mastering the growth distribution of *C. oleifera*, rapid yield measurement, and achieving accurate management of *C. oleifera* forests, accurate and efficient acquisition of *C. oleifera* crown information is crucial (Yan et al., 2021; Ji et al., 2022).

In recent years, UAV remote sensing technology with a visible digital camera has become one of the most important methods for obtaining crop growth data due to its high resolution, real-time, and adaptability, allowing for the efficient acquisition of high-precision tree-crown data (Wang et al., 2004; Dash et al., 2019; Pearse et al., 2020; Shu et al., 2021). For crown information extraction, object-oriented classification (Zhang et al., 2015; Wu et al., 2021), watershed (Imangholiloo et al., 2019; Wu et al., 2021), local maximum (Lamar et al., 2005), and region-growing method (Pouliot and King, 2005; Bunting and Lucas, 2006) are often used. These techniques have yielded successful crown detection results for pure forests, plantations, or specific tree species and images. However, image processing parameter settings are too dependent on expert knowledge (Chadwick et al., 2020), making it difficult to automatically extract image information (Laurin et al., 2019). Therefore, new methods are required to rapidly extract tree-crown data to improve tree growth monitoring.

In recent years, image segmentation techniques based on deep learning technology that can automate and batch-process data have been widely adopted (Li et al., 2016; Kattenborn et al., 2021). Among them, the U-Net network based on Fully Convolutional Network (FCN) focuses more on segmentation details due to its capability of feature stitching and multi-scale fusion, which performs well in image segmentation (Ronneberger et al., 2015). In forestry, the U-net model has been applied successfully to tasks such as extracting tree canopy information from UAV imagery. Li et al. (2019) extracted the crown of the poplar with an accuracy of 94.1% using the U-Net network. However, if the number of U-Net network layers is excessive, network degradation will occur, and segmentation accuracy will decrease (Yang et al., 2020). The unique residual structure of the residual network can effectively mitigate the network degradation problem caused by the deep network structure and speed up network convergence (He et al., 2016). ResU-Net, which is created by combining Res-Net and U-Net, can include more layers and prevent model performance degradation (Ghorbanzadeh et al., 2021). Tong and Xu (2021) fused ResNet-34 and U-Net convolutional neural networks to create the ResNet-UNet (ResU-Net) stumpage segmentation model, improving accuracy and robustness significantly. However, the research mentioned above focuses primarily on macrophanerophytes, and there are fewer studies on the extraction of *C. oleifera* crown parameters. In addition, when detecting tree crowns, images with only three bands (red, green, and blue) are typically used as model input images (Neupane et al., 2019; Weinstein et al., 2019). Few studies have used multi-band images with elevation data (digital surface and canopy height models) as input images to train models for detecting tree-crown and estimating tree-crown width and projection area.

According to this context, this study is the first to apply ResU-Net to *C. oleifera* tree-crown extraction. UAV imagery with added elevation information (DSM and CHM) is used to create ResU-Net training datasets. This study investigates the capability of the semantic segmentation model ResU-Net to extract *C. oleifera* crowns from multi-band combined images with elevation information derived from UAV imagery. This study aims to (1) propose combined images with elevation information for a ResU-Net model to detect the tree-crown information of *C. oleifera* and (2) evaluate the models trained using various image combinations and select the optimal model for practical applications. This study is expected to provide more precise data support for the extraction of *C. oleifera* tree-crown information to better monitor and manage non-wood forests.

## Materials and methods

**Figure 1:**
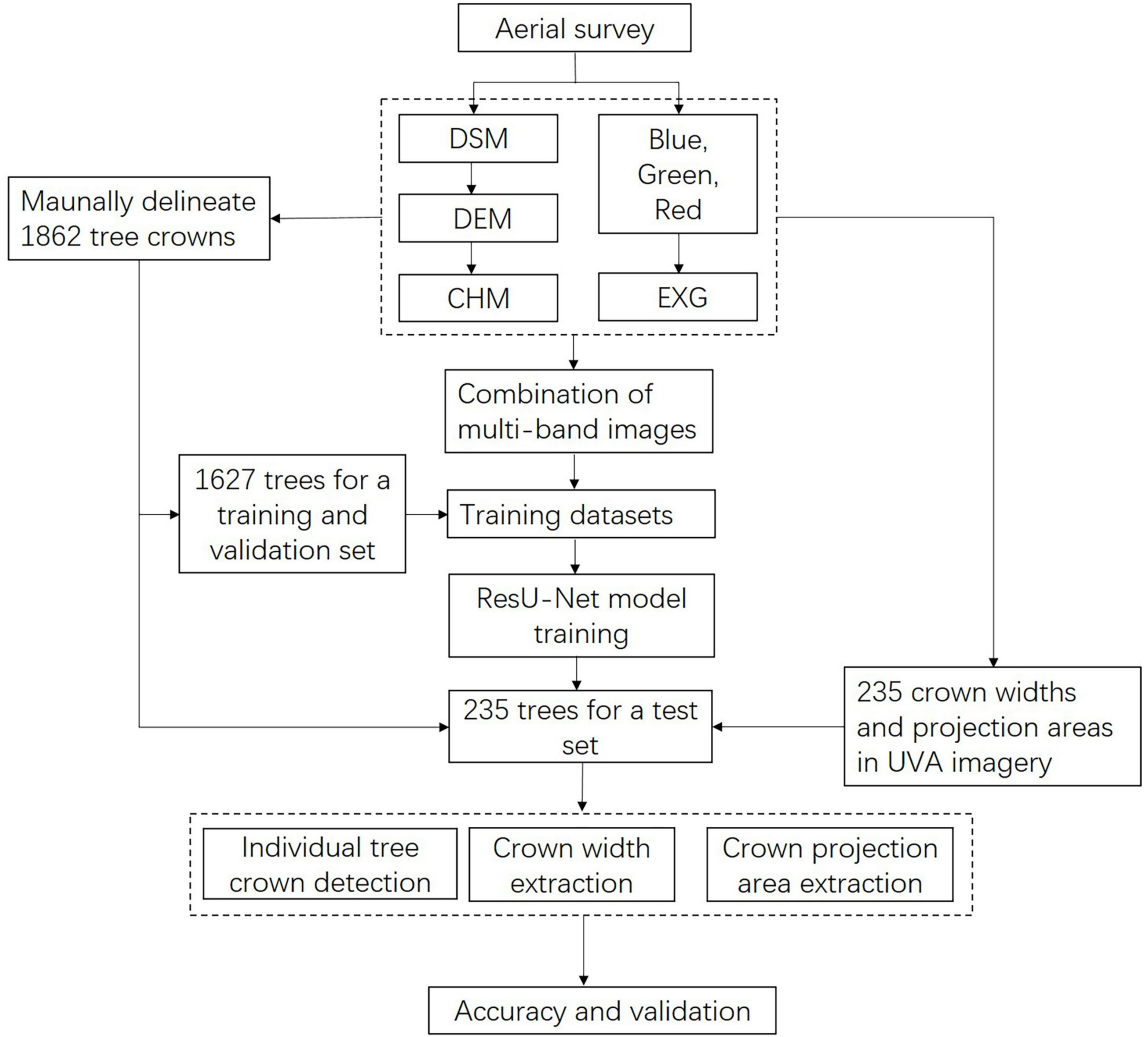
Flowchart of individual tree-crown detection, crown width, and projection area assessment in this study. DSM, digital surface model; DEM, digital elevation model; CHM, canopy height model; EXG, excess green index.

Figure 1 illustrates the framework of this study. As input images, six distinct band-combined images were created first. The input and tree-crown binary images are split and amplified to obtain the training data set. The training dataset is utilized for the training of the proposed model. Then, six distinct ResU-Net models were utilized to estimate the number of plants, crown width, and projection areas at the study site. The model’s performance was then evaluated, including the precision of individual tree-crown detection, crown width, and crown projection area estimation.

### Study site

**Figure 2:**
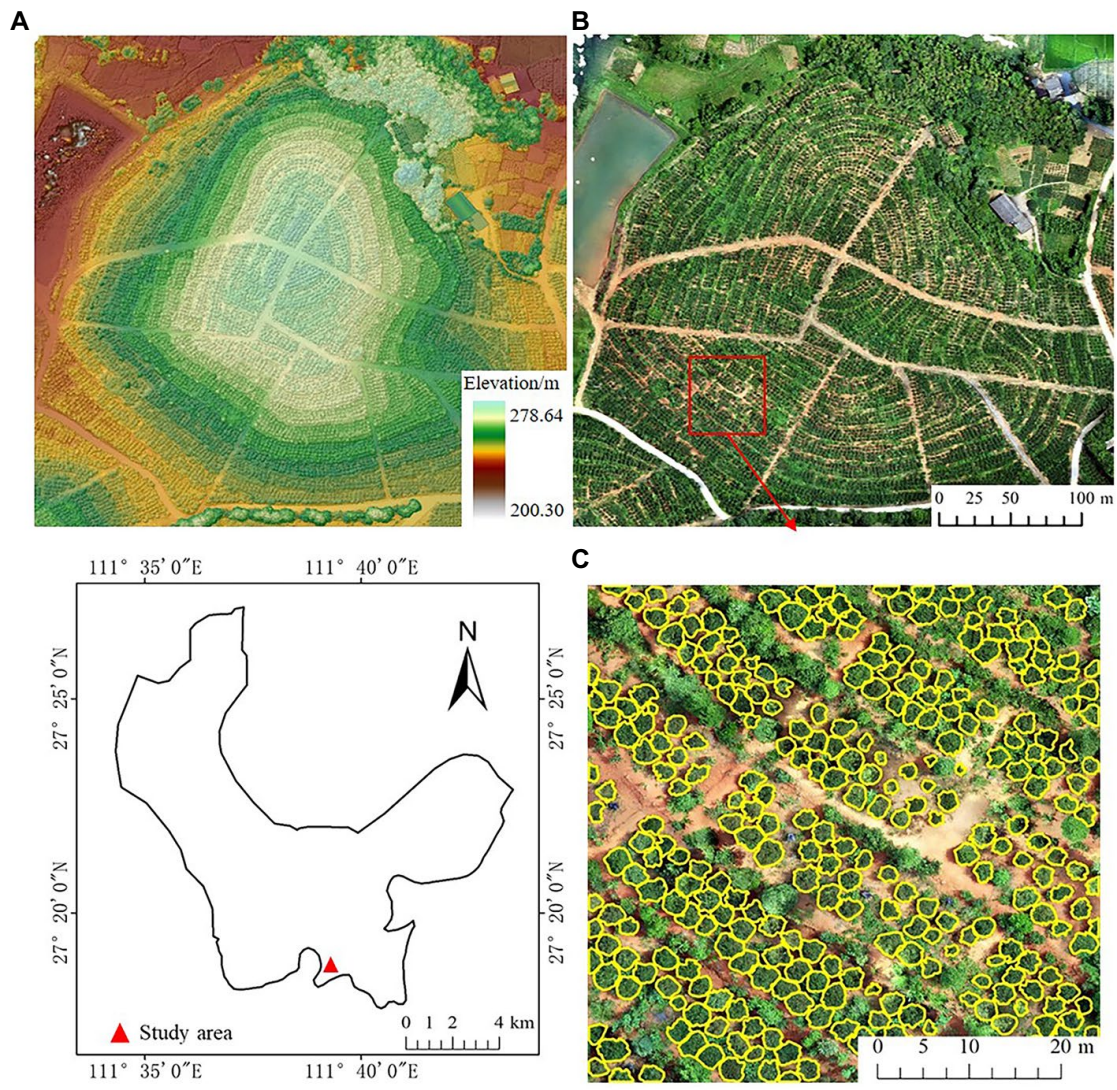
Location of the study site, Chengiafang County, Hunan, China; **(A)** digital surface model (DSM); **(B)** orthomosaic with RGB bands; **(C)** example of manually delineated *Camellia oleifera* crowns (in yellow) based on orthomosaic.

The study site is located in Chenjiafang Town, Xinshao County, Shaoyang City, Hu-nan Province, between 111°08′–111°05′E and 27°15–27°38′N (Figure 2). The region has a humid mid-subtropical continental monsoon climate and average annual precipitation of 1365.2 mm, making it a typical low-hilly terrain in the south. *Camellia oleifera* was planted in the study area on a total area of 59.18 hm^2^ in 2014.

### Data collection and preprocessing

#### Image acquisition

The UAV imagery was collected using Phantom 4 RTK^1^ on July 4, 2021, in diffuse light weather to avoid the influence the tree shadows have on the aerial survey results. The UAV is equipped with a 1-inch COMS sensor. The focal length of the sensor is 24 mm, the aperture range is f/2.8–f/11, and the image resolution of the camera is 5,472 × 3,648 pixels (JPEG format). The flight altitude was set to 40 m, the course speed was 3 m/s, the bypass overlap rate was set to 70%, the heading overlap rate was set to 80%, and a total of 1,127 images were captured. The UAV imagery was pre-processed using Agisoft Metashape 1.7.1 software from Agisoft LLC, Russia, which generated the digital surface model (DSM) and the RGB-banded orthomosaic. The DSM is minimally filtered (window size is 20 × 20) and then smoothed with a mean filter (window size is 5 × 5) to generate the digital elevation model (DEM), which is then subtracted from the DSM to generate the canopy height model (CHM).

The objects in the UAV imagery are primarily plants (green in color) and backgrounds (soil, rocks, plant debris, etc., which are primarily earthy in color), so the red, green, and blue bands of orthomosaic are calculated to generate EXG images (Equation 1), which are used to highlight green plants and suppress backgrounds such as shadows, rocks, and soil (Woebbecke et al., 1995).


(1) EXG=2G−R−B

Where R, G, and B are the three standard bands of red, green, and blue, respectively.

#### Field survey data

Using UAV imagery, select 235 *C. oleifera* trees randomly and determine their exact location. Utilize a measuring rod to determine the height of the trees in the study area. The method is feasible because the height of *C. oleifera* is limited (<3 m), the tops of the trees are visible, and the distance between trees is known.

#### Tree-crown delineation

The tree crowns of *C. oleifera* were manually outlined in ArcMap 10.7 (ESRI, United States) using orthomosaic and CHM data (Table 1). There were a total of 1,862 crowns outlined (Figure 2C). Then, the manually delineated tree-crown image is binarized, the background pixel value is changed to 0, the pixel value of the *C. oleifera* crown area is changed to 255, and a tree-crown binary image is generated that corresponds to the tree-crown in the UAV image.

**Table 1:** Statistics of crown width and crown projection area.

Item	CW/m	CAP/m^2^
min	1.16	1.40
max	3.47	5.58
mean	1.99	2.95

CW, crown width; CAP, crown projection area.

The CV2 function provided by OpenCV, an open-source computer vision library, was used to count the number of pixels contained in the tree-crown of each of the 235 trees (Section “Field survey data”) based on the tree-crown mask image and calculate the crown projection area (CPA) based on the image resolution. Using Canny’s algorithm (Hu et al., 2018) to extract the edge features of each crown of 235 trees, followed by the ellipse fitting algorithm (Yan et al., 2008) to obtain each crown’s external ellipse. Calculate the long and short axes of the ellipse as the maximum and minimum values of *C. oleifera* crown width, and then calculate the mean of these two values to obtain the average crown width of *C. oleifera* (Zhang et al., 2021). The crown width and projection area of 235 trees were calculated based on the 0.01532703 m image resolution.

#### Dataset preparation

##### Input image

The blue, green, red, and EXG products are used as input images for the division of the tree crown. The CHM or DSM was added to the combined images to compare the effects of input images containing different elevation information on the model’s ability to accurately estimate the crown width and projection area (Table 2).

**Table 2:** Combination of different images for ResU-Net training.

Combinations	Bands description	Image layers
RGB	Blue, green, red	3
RGB-DSM	Blue, green, red, DSM	4
RGB-CHM	Blue, green, red, CHM	4
EXG	Excess green index	1
EXG-DSM	Excess green index, DSM	2
EXG-CHM	Excess green index, CHM	2

##### Training dataset

Thousand eight hundred and sixty-two delineated trees (Section “Tree crown delineation”) were separated into a training and validation set and a test set for this study. First, the six-band combination images containing information about the tree crowns of 1,627 trees and the corresponding binary tree-crown images are divided into 256 × 256 pixel image tiles for processing. In addition, the training data are rotated by 90°, 180°, and 270° from its original orientation to increase the number of training samples and enhance the model’s robustness. In summary, six training datasets, each one containing 3,375 images, were obtained for this study.

To evaluate the performance of the final model, 235 *C. oleifera* trees with crown width and projection area reference information from UAV imagery were used as the test set.

### ResU-Net model

**Figure 3:**
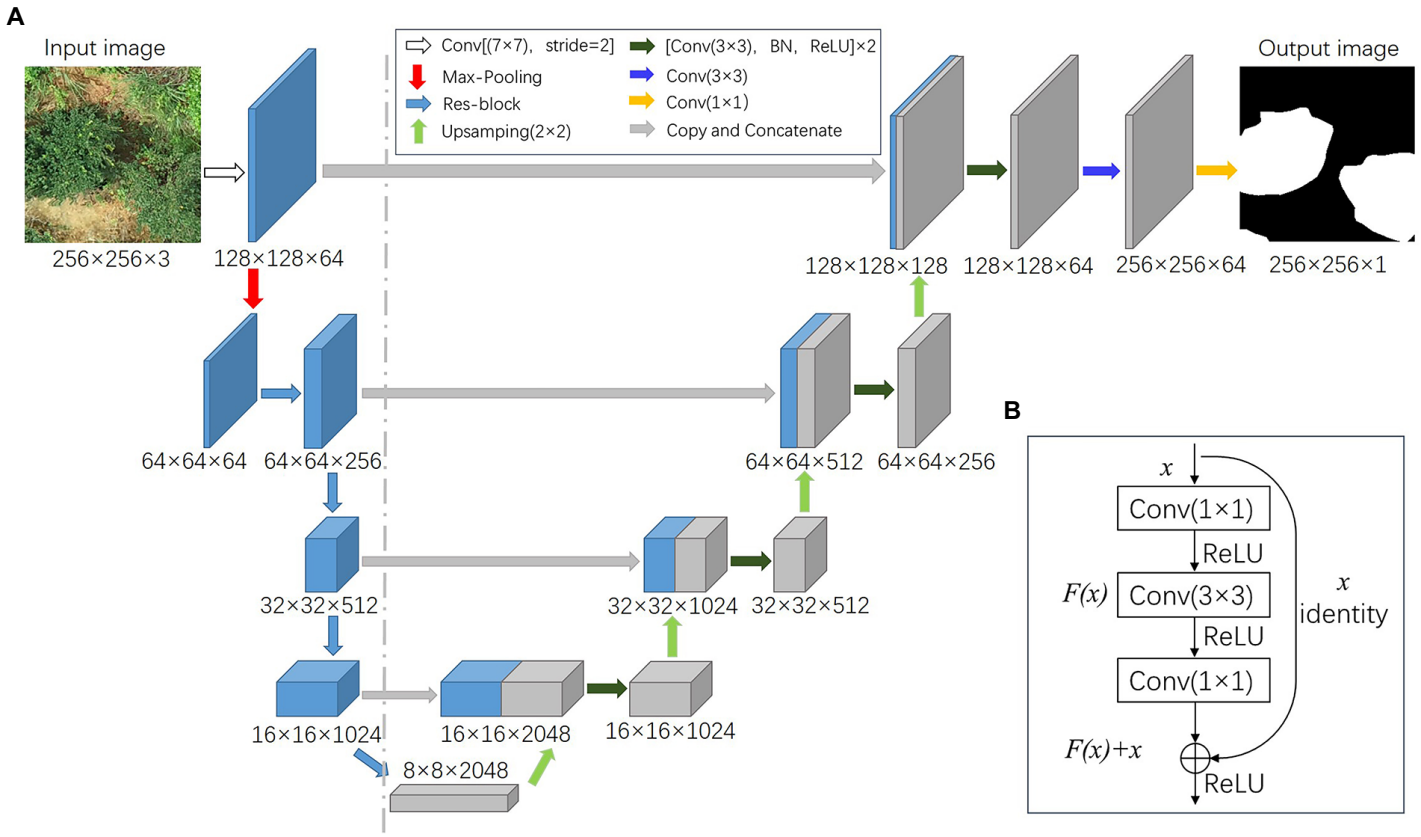
ResU-Net network structure: **(A)** ResU-Net network framework; **(B)** Res-block modules.

Since *C. oleifera* tree-crown images contain a large number of background interference factors, such as weeds and soil, ResNet101, which has a strong feature extraction capability (Laurin et al., 2019), is used as the backbone network and combined with the U-Net (Ronneberger et al., 2015) network design concept to create the ResU-Net network model in this study. ResU-Net presents the Residual Block (Res-block) structure (illustrated in Figure 3B) based on the U-Net network, which can effectively overcome the network degradation and gradient dispersion issues caused by an increase in network depth and accelerate network convergence (Wu et al., 2019). ResU-Net requires a smaller training set and focuses more on image segmentation details without compromising accuracy (Yang et al., 2019), and it can recognize the crown of *C. oleifera* at the pixel level.

The encoder (to the left of the dashed line) and decoder (right of the dashed line) make up the ResU-Net structure, as shown in Figure 3A. The encoder is used to downsample the input image, capture image context information, and extract image semantic information features. The decoder upsamples the image using transposed convolution and concatenates features of the same dimension to provide detailed feature information (Chen et al., 2021).

For the encoder, the input image passes through a Convolution layer (CONV) with a convolution kernel of 7 × 7 and a step size of 2, followed by a Max Pooling Layer. The image size is decreased to a quarter of its original size, and the number of channels is increased to 64. Following this, the tree-crown features are extracted through the residual block (Res-block) until an 8 × 8 feature vector with a depth of 2048 is obtained.

The initial step for the decoder is to increase the size of the feature layer and decrease its depth by upsampling. Next, the downsampled and upsampled feature layers of equal size are concatenated. The concatenated feature layers are fused using a 3 × 3 convolutional layer, a Batch Normalization layer (BN), and a Rectified linear unit after each concatenation (ReLU). The tree-crown binary mask of *C. oleifera* is obtained through a final convolution operation.

The hold-out method is selected for cross-validation, and the mIoU of the model is set to stop in advance without increasing within 10 epochs to prevent overfitting of the model. Only the model with improved accuracy after each training is saved. Res-Net weights pre-trained on ImageNet are used for transfer learning. The ResU-Net model was then trained using the six training datasets. Set the learning rate parameter to 0.001, the epochs to 100, and the batch size to 4 when training the model. Based on these six distinct composite images, six ResU-Net models were generated. All models were trained on a Windows 10 desktop with an Intel i7 6700k CPU, 6 GB GPU, and 24 GB RAM using PyCharm 2010.1.4 software based on the Pytorch framework for deep learning.

### Accuracy evaluation

To determine the optimal ResU-Net model, the accuracy of tree detection, crown width, and projection area assessment were calculated separately. The test set, which contained crown width and projection area reference data from the UAV images, was utilized to assess the performance of each model.

The intersection over union (IoU) was used to assess the ResU-Net model’s accuracy in detecting tree crowns (Equation 2). IoU measures the area of the union and intersection of the crown polygons of the test set and the crown polygons predicted by the model. When IoU exceeded 50%, it was deemed acceptable. ResU-Net model’s detection of individual tree crowns was evaluated using precision, recall, and F1 score (Equations 3–5). Precision is the proportion of accurately detected trees in a model detection. The recall is the proportion of correctly identified trees in the test set. The F1 score indicates the overall test accuracy, which is based on recall and precision (Shao et al., 2019; Hao et al., 2021).


(2) $$\mathrm{IoU}=\frac{A_{\mathrm{actual}}\cap A_{\mathrm{predicted}}}{\;A_{\mathrm{actual}}\cup A_{\mathrm{predicted}}}\times 100\%$$

(3) $$P=\frac{\mathrm{TP}}{\mathrm{TP}+\mathrm{FP}}\times 100\%$$

(4) $$R=\frac{\mathrm{TP}}{\mathrm{TP}+\mathrm{FN}}\times 100\%$$

(5) $$F_{1}=\frac{2PR}{P+R}\times 100\%$$

where TP is the number of correctly identified trees by the model and IoU is >50%. FN is the number of trees omitted by the model when the IoU is <50%, and FP is the number of other tree or weed species found. The term Aactual refers to the crown polygons of the test set. The crown polygons predicted by the ResU-Net model are indicated by Apredicted. The intersection operation represents the area shared by Aactual and Apredicted, whereas the union operation represents the area formed when Aactual and Apredicted are combined.

The 235 tree-crown widths and projection area from the UAV imagery were then compared to the six ResU-Net model predictions. Coefficient of determination (*R*^2^), root mean square error (RMSE), and relative RMSE (rRMSE) were utilized to evaluate the model’s accuracy in estimating tree-crown width and projection area (Equations 6–8). *R*^2^ is utilized to assess fitness, while RMSE and rRMSE are employed to estimate error.

Finally, a comprehensive analysis of the accuracy of individual tree-crown detection, crown width, and predicted area assessment was conducted to determine the best ResU-Net model for crown width and crown projection area assessment.


(6) $$R^{2}=1-\frac{\sum_{i=1}^{N}{\left(y_{i}-x_{i}\right)}^{2}}{\sum_{i=1}^{N}{\left(y_{i}-{\bar{y}}_{i}\right)}^{2}}$$

(7) $$\text{RMSE}=\sqrt{\frac{\sum_{i=1}^{N}{\left(y_{i}-x_{i}\right)}^{2}}{N}}$$

(8) $$r\mathrm{RMSE}=\frac{\mathrm{RMSE}}{\bar{x_{i}}}$$

where *N* represents the number of trees that the model has detected. *y*_i_ denotes the crown width and predicted area from the assessed datasets. ${\bar{y}}_{i}$ represents the average crown width and predicted area from the assessed datasets; *x*_i_ denotes the crown width and predicted area from the reference dataset; and ${\bar{x}}_{i}$ represents the average crown width and predicted area from the reference dataset.

## Results

### Detection of individual tree crown

**Figure 4:**
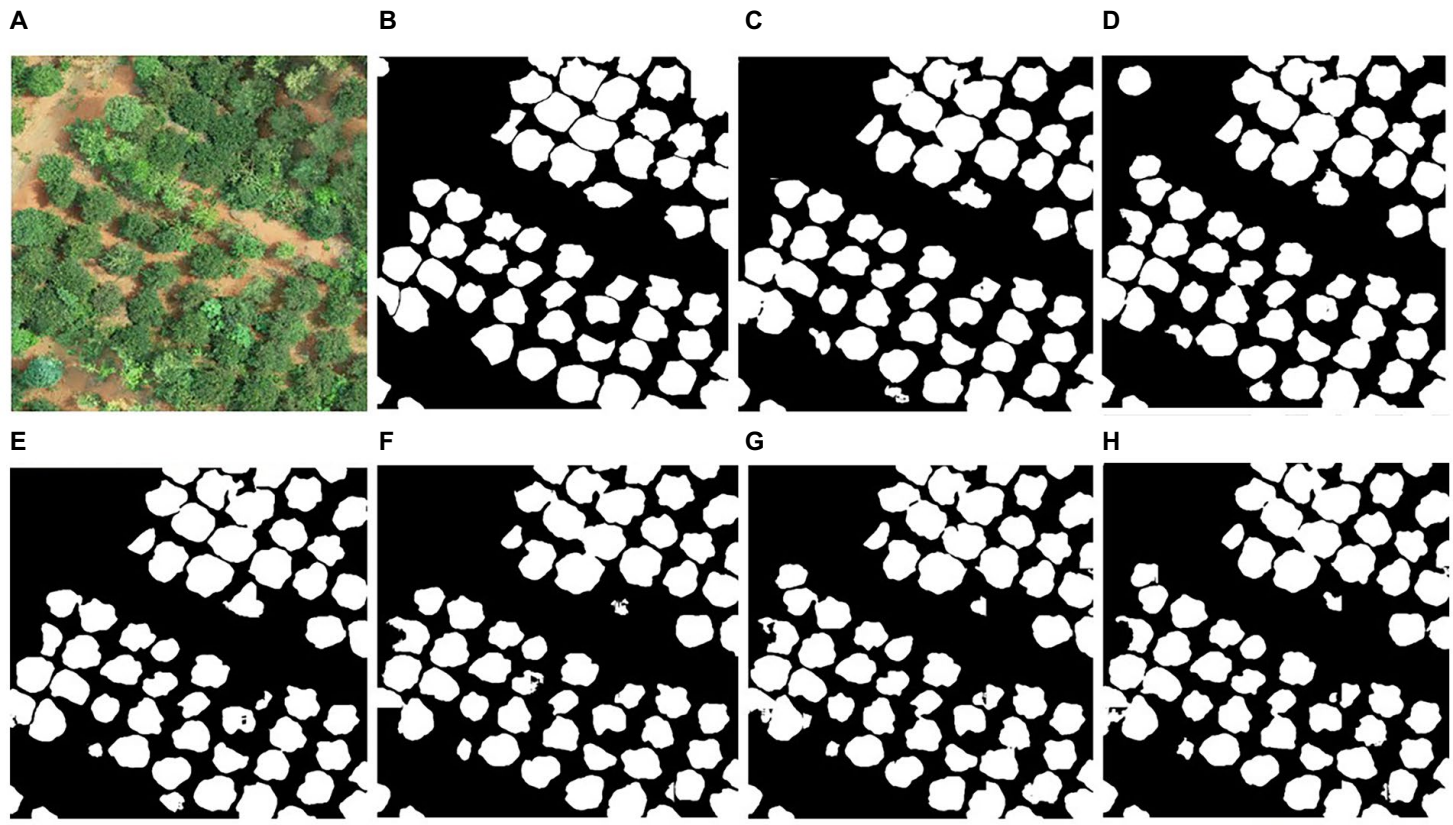
Example of tree-crown detection and segmentation. **(A)** original image; **(B)** manually delineated result; **(C–H)** are the crown detection results of the ResU-Net model based on the combination of RGB, RGB-CHM, RGB-DSM, EXG, EXG-CHM, and EXG-DSM.

Figure 4 illustrates examples of the ResU-Net model identifying individual tree crowns at the study site. The accuracy of crown detection and delineation is shown in Table 3. RGB-CHM had the highest precision (88.73%) for tree detection, followed by RGB-DSM (87.92%), RGB (85.00%), EXG-CHM (82.30%), EXG-DSM (80.71%), and EXG had the lowest precision (80.68%). The RGB-CHM combination had the highest recall rate (80.43%), while the EXG-DSM combination had the lowest (67.66%). The F1 score of the RGB-CHM combination reached 84.38%, followed by RGB-DSM (82.35%), RGB (82.20%), EXG-CHM (77.48%), EXG (75.57%), and EXG-DSM (73.61%). The RGB-CHM combination had the highest IoU (91.38%) for tree-crown delineation, while the EXG-DSM combination had the lowest (87.07%).

**Table 3:** The accuracy of individual tree-crown detection using different combinations.

Bands combinations	Estimate number	Correct number	Incorrect number	Omission number	IOU /%	Precision /%	Recall /%	F1 score /%
RGB	220	187	33	48	91.06	85	79.57	82.2
RGB-CHM	213	189	24	46	91.38	88.73	80.43	84.38
RGB-DSM	207	182	25	53	89.86	87.92	77.45	82.35
EXG	207	167	40	68	87.07	80.68	71.06	75.57
EXG-CHM	209	172	37	63	88.48	82.3	73.19	77.48
EXG-DSM	197	159	38	76	87.09	80.71	67.66	73.61

The results of all ResU-Net models yielded precision >80%, recall >65%, F1 score >70%, and IoU >85%. When the input image is based on the RGB or EXG combinations of ResU-Net and contains CHM, the precision, recall, F1 score, and IoU are greater than when the input image contains DSM, reflecting the higher precision of tree-crown detection and delineation in the CHM combination. The model’s average processing time for tree-crown detection in each image is 0.16 s, which meets the requirements of practical applications.

### Extraction of tree-crown width

**Figure 5:**
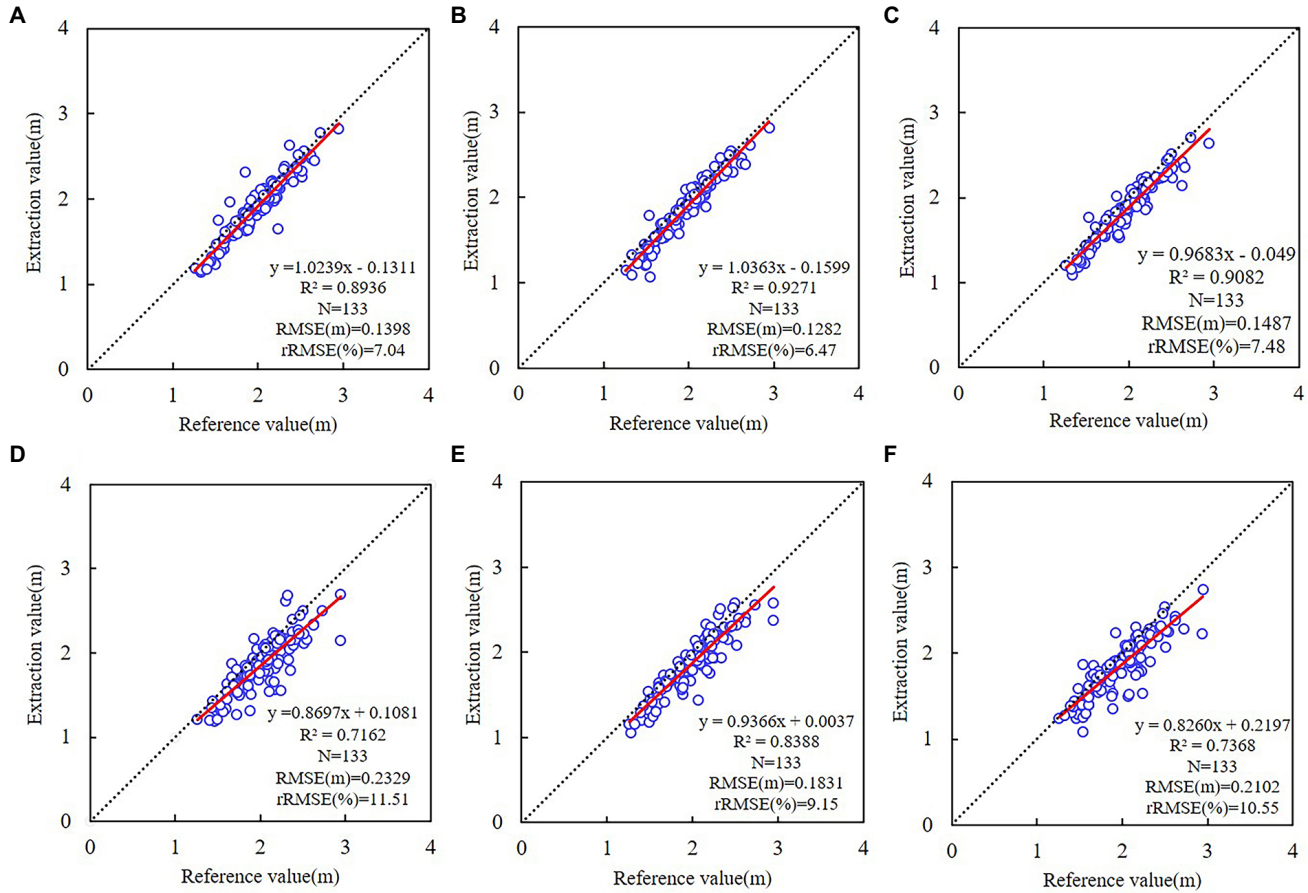
Linear regressions of tree-crown width between UAV imagery and different ResU-Net models. **(A)** RGB; **(B)** RGB-CHM; **(C)** RGB-DSM; **(D)** EXG; **(E)** EXG-CHM; **(F)** EXG-DSM. The dotted line represents a 1:1 match, and the red line represents the trend of the tree-crown width relationship based on the ResU-Net model and UAV imagery.

Using the UAV-based tree-crown width as reference. As a result of their proximity to the study area’s boundaries, certain canopies were omitted from the evaluation of the model’s accuracy in estimating tree-crown width, resulting in incomplete shapes. The accuracy of the ResU-Net model in estimating the tree-crown width based on RGB, RGB-CHM, RGB-DSM, EXG, EXG-CHM, and EXG-DSM as input images, respectively, is illustrated in Figure 5.

All the results of the six models for estimating tree-crown width yielded *R*^2^ > 0.70. The tree-crown width estimation based on the RGB combinations yielded higher accuracy (*R*^2^ > 0.89, 6.47% ≤ rRMSE ≤ 7.48%). The accuracy based on the EXG combinations is lower (*R*^2^ < 0.84, 9.15% ≤ rRMSE ≤ 11.51%). Among them, the accuracy of estimating tree-crown width using the DSM combination was the lowest, while the CHM combination produced better results, and the RGB-CHM combination achieved the highest accuracy (*R*^2^ = 0.9271, RMSE = 0.1282 m, rRMSE = 6.47%).

### Extraction of tree-crown projection area

**Figure 6:**
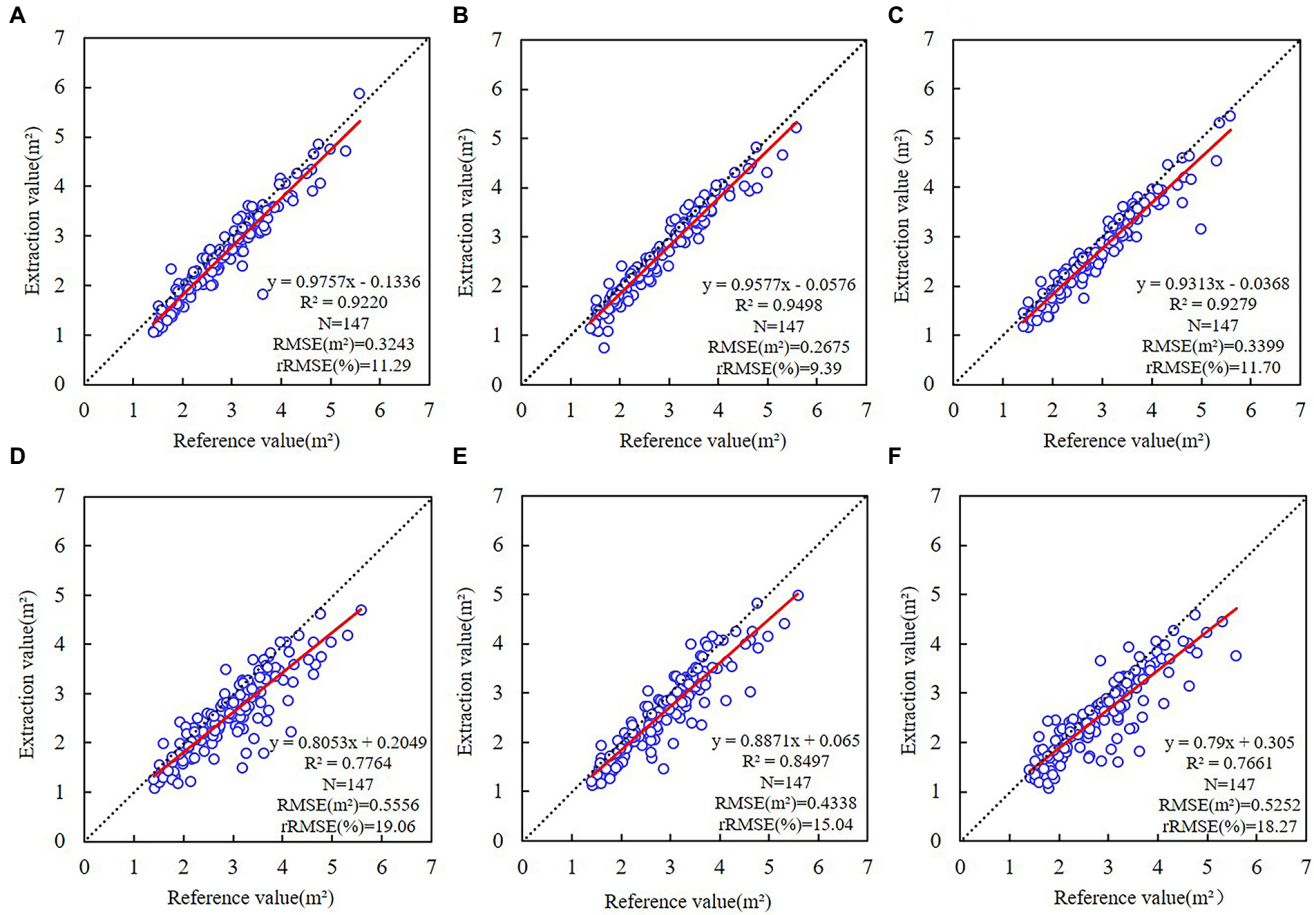
Linear regressions of crown projection area between UAV imagery and different ResU-Net models. **(A)** RGB; **(B)** RGB-CHM; **(C)** RGB-DSM; **(D)** EXG; **(E)** EXG-CHM; **(F)** EXG-DSM. The dotted line represents a 1:1 match, and the red line represents the trend of the crown projection area relationship based on the ResU-Net model and UAV imagery.

The projected tree-crown area estimated by six distinct ResU-Net models was compared to the UAV-based crown projection area (Figure 6). The accuracy of tree-crown projection area estimation based on the RGB combinations (*R*^2^ > 0.92, 9.39% ≤ rRMSE ≤ 11.70%) was higher than that based on the EXG combinations (*R*^2^ < 0.85, 15.04% ≤ rRMSE ≤ 19.06%). Among them, the CHM model was more accurate than the DSM model. The model with the RGB-CHM combination produced the highest accuracy (*R*^2^ = 0.9498, RMSE = 0.2675 m^2^, rRMSE = 9.39%), while the model with the EXG combination produced the lowest accuracy, which is comparable to the tree-crown width from the UAV imagery. ResU-Net model predictions were lower compared to the UAV-based crown width and crown projection area because of the overlapping and shading conditions with unclear boundaries.

The ResU-Net model accurately estimated the tree-crown width and crown projection area. RGB was more accurate than EXG when it came to modeling accuracy. The accuracy is greater when the input image of ResU-Net-based RGB or EXG combinations contains CHM than when the input image contains DSM. Comparing the accuracy of individual tree-crown detection, tree-crown width, and projection area between models, the RGB-CHM combination was the optimal combination for the ResU-Net model’s detection of tree-crown width and projection area.

## Discussion

This study proposes to use the combined images and elevation data from UAV imagery to create datasets for training ResU-Net models to automatically extract *C. oleifera* tree-crown and estimate crown width and crown projection area parameters. The results demonstrate that the trained ResU-Net model can detect tree crowns and accurately estimate crown width and crown projection area. The ResU-Net model has excellent generalizability and high stability, allowing it to fulfill the need for *C. oleifera* tree-crown data in agricultural production.

### Performance of the model

#### Individual tree-crown detection

The optimal ResU-Net model based on the RGB-CHM combination achieved high accuracy for tree-crown detection (precision = 88.73%, recall = 80.43%, F1 score = 84.68%, and IoU = 91.38%). Hao et al. (2021) and Braga et al. (2020) used the Mask-RCNN model to detect macrophanerophyte canopies, yielding F1scores of 84.68% and 86%, which are comparable to the F1-score of this study, whereas the IoU values yielded 91.27% and 61% are smaller than the IoU of this study, respectively. Jin et al. (2020) reported that the F1-score of 74.04% and accuracy of 79.45% for tree-crown detection based on U-Net and marker-control watershed algorithm, which is lower than the F1 score and accuracy of this study.

**Figure 7:**
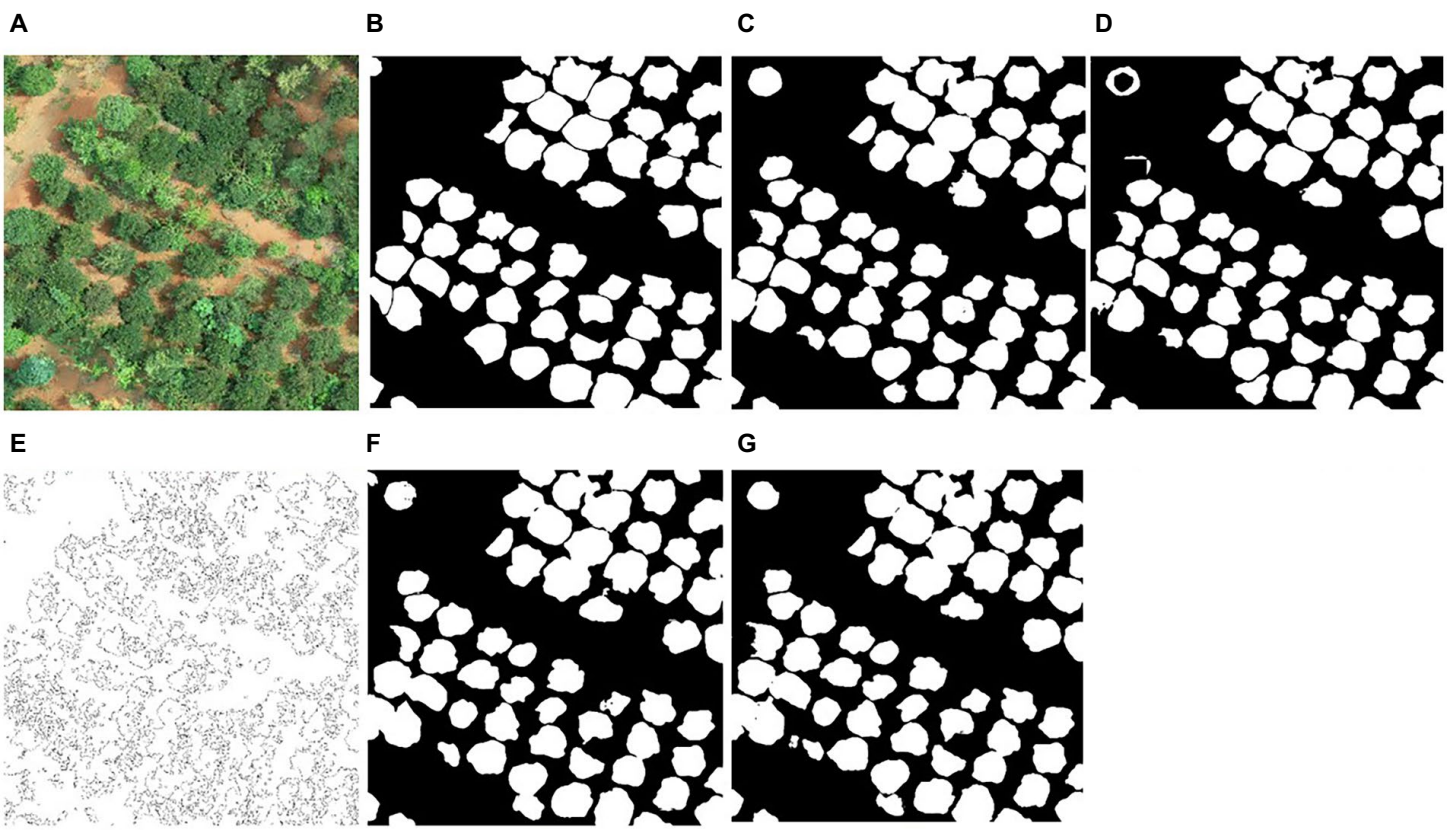
Comparison of tree-crown detection results between ResU-Net and other methods. **(A)** original image; **(B)** manually delineated result; **(C–G)** are examples of tree-crown detection using the ResU-Net model, DeepLabV3+ model, watershed algorithm, U-Net model, and U-Net++ model.

Next, this study compares the performance of the ResU-Net with the classical watershed algorithm, the U-Net model, the U-Net++ model (Zhou et al., 2018), and the DeepLabV3 Plus model (Chen et al., 2018) for tree-crown detection (Figure 7). According to the crown detection results, the crown detection accuracy based on the deep learning method is significantly higher than that of the classical watershed algorithm. Since *C. oleifera* trees are lower and there is interference from grass and other tree species, etc., the watershed algorithm is less precise. The accuracy of tree-crown detection using the ResU-Net model was slightly higher than that of the U-Net (precision = 86.18%, recall = 79.57%, F1 score = 82.74%, and IoU = 90.75%) and U-Net++ model (precision = 87.50%, recall = 77.45%, F1 score = 82.17%, and IoU = 90.96%). Using the DeepLabV3 Plus model for tree-crown detection yielded the accuracy (precision = 89.41%, recall = 79.72%, F1 score = 84.29%, and IoU = 91.85%) comparable to the ResU-Net model. In conclusion, the detection accuracy of different network models for tree crowns is similar, indicating that the application of deep learning methods for extracting *C. oleifera* tree crowns from UAV visible images is universal, and the accuracy is generally high. With the advancement of deep learning techniques, we can utilize more robust network models for *C. oleifera* tree-crown detection in the future study.

#### Crown width assessment

The key innovation of this study is to use the ResU-Net model and combine images with elevation data to estimate crown width. The RGB-CHM combination provided the most accurate measurements of crown width (*R*^2^ = 0.9271, RMSE = 0.1282 m, rRMSE = 6.47%). Few studies combine the ResU-Net model with elevation data to estimate the crown width of *C. oleifera*. Consequently, the accuracy of crown width and projection area estimation in our study is compared to that of studies employing alternative remote sensing techniques. Wu et al. (2021) used the optimized watershed with multi-scale markers method to estimate *C. oleifera* crown width yielded *R*^2^ = 0.75. Dong et al. (2020) estimated the crown width of apple and pear trees using local maximum and marker-controlled watershed algorithms, yielding *R*^2^ values of 0.78 and 0.68, respectively. Compared to these conventional remote sensing techniques (e.g., watershed, local maximum algorithms), the present method has greater accuracy in estimating crown width, and it can be automated.

#### Crown projection area assessment

The RGB-CHM combination provided the most accurate measurements of crown projection area (*R*^2^ = 0.9498, RMSE = 0.2675 m^2^, and rRMSE = 9.39%). Mu et al. (2018) estimated the peach crown projection area using adaptive thresholding and marker-controlled watershed segmentation with *R*^2^ = 0.89 and RMSE = 3.87 m^2^. Dong et al. (2020) estimated the crown projection area of apple and pear trees using local maximum and marker-controlled watershed algorithms, yielding *R*^2^ values of 0.87 and 0.81, respectively. Ye et al. (2022) estimated the olive crown projection area using the U^2^-Net model, producing *R*^2^ > 0.93, which is comparable to our study. However, it yielded MRE = 14.27% higher than our study (MRE = 12.23%).

Next, compared to the crown projection area extracted using RGB (*R*^2^ = 0.9220), *R*^2^ increased by 0.0278 with the addition of CHM, and by 0.0059 with the addition of DSM, respectively. Compared to the crown width extracted using RGB (*R*^2^ = 0.8936), *R*^2^ increased by 0.0335 with the addition of CHM, and by 0.0146 with the addition of DSM, respectively. As can be seen, the addition of CHM to the model has a greater impact on the prediction accuracy of the CW and CPA than the addition of DSM. However, the increased value (<0.05) of the model accuracy after adding the elevation information is low, because the *C. oleifera* planting areas are mainly hilly with little elevation change, and the *C. oleifera* are shrubs with low tree height. To verify the reliability of the experimental results of this study, it is necessary to conduct in-depth experiments in a region with a large height difference in the future study.

In addition, this study discovered that the accuracy of the EXG combinations was lower than that of the RGB combinations, indicating that the limited features of EXG (grayscale maps) reduce the model’s accuracy (Hao et al., 2021). Therefore, it is recommended that adequate features are included in the training process.

### Factors of influence on model performance

#### Accuracy of CHM extraction

The accuracy of canopy height model (CHM) extraction influences the model-detected tree crown, the estimated crown width, and the crown projection area. To evaluate the accuracy of CHM, the UAV-derived tree height estimates were compared with field measurements in this study.

**Figure 8:**
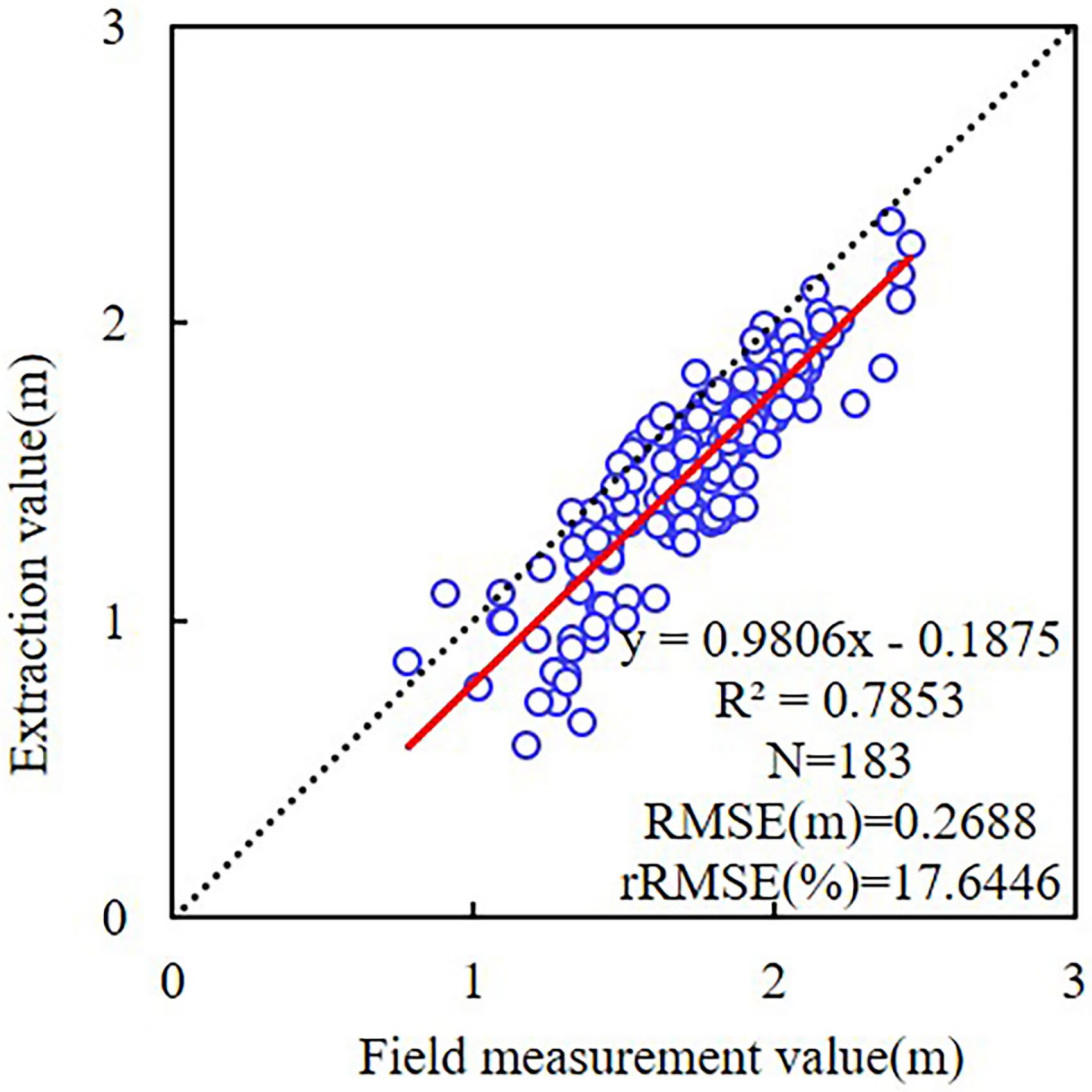
Linear regressions of tree height between the field measurement and different ResU-Net models.

In this study, the CHM and the manually outlined tree-crown vector map were combined. Then, ArcMap 10.7 was utilized to extract the maximum value of CHM for each tree-crown region and estimate the tree height from the UAV imagery. For the accuracy of the results, incomplete *C. oleifera* crowns at the image margins were omitted. There were 183 complete canopies that corresponded to the estimated tree height values when measured on the ground. As depicted in Figure 8, the correlation between the estimated tree height and field measurements was strong, with *R*^2^ of 0.7853, RMSE of 0.2688 m, and rRMSE of 17.64%. The estimated tree heights were lower than field measurements, likely due to the high density of *C. oleifera* forests and lack of bare ground, which prevented the filtering method from obtaining sufficient ground area when DSM was processed. Consequently, the digital elevation model (DEM) is higher than the true elevation value, whereas the CHM is lower than the true CHM.

Airborne laser scanning (ALS) has a high penetrability and is considered the best option for tree detection with high accuracy in dense canopy areas (Sankey et al., 2017; Pourshamsi et al., 2021). Therefore, orthophotos and ALS can be combined as input data when training the model.

#### Spatial image resolution

The spatial resolution of the image has a significant impact on the accuracy of the model’s detection of tree crowns. By comparing 0.3, 1.5, 2.7, and 6.3 cm spatial resolutions, Fromm et al. (2019) determined that the model had the highest average accuracy for detecting conifer seedlings when the spatial resolution was 0.3 cm. Schiefer et al. (2020) concluded that, when using five spatial resolution images for crown detection, the lower the spatial resolution, the lower the model detection accuracy. Studies have demonstrated that the higher the spatial resolution, the more precise the model’s crown detection. However, when the resolution exceeds a certain threshold, it does not improve the accuracy of models significantly (Hao et al., 2021). The model’s accuracy is also dependent on the size of the tree crowns, and the ratio of the tree-crown diameter to the spatial resolution is a crucial factor in determining the detection accuracy. Yin and Wang (2019) suggested that images with a spatial resolution greater than a quarter of the crown diameter had the highest accuracy for crown detection; however, 0.25 m resolution had the highest accuracy for mangrove crowns compared to 0.1, 0.5, and 1 m resolutions. An excessively high spatial resolution will generate interference due to excessive detail and noise, which is not conducive to model crown detection.

In addition, the measurement error resulting from manual delineation and the calculation error of crown parameters will also have an impact on the model estimation results. For optimal tree-crown extraction results, the measurement method must be optimized. The ratio relationship between spatial image resolution and tree-crown size must be further investigated in a subsequent study.

## Conclusion

Combining the ResU-Net model with images that add elevation information (CHM or DSM) from UAV imagery can effectively and automatically detect *C. oleifera* tree crowns and estimate the crown width and crown projection area, which has significant application potential. ResU-Net model with RGB-CHM combination outperformed other models with different combinations (Precision = 88.73%, IoU = 91.38%, Recall = 80.43%, and F1 score = 84.38%). The model’s accuracy using RGB combinations was superior to the model’s accuracy using EXG combinations. The accuracy of crown width and crown projection area estimation is dependent on the input elevation data (DSM or CHM), and the model with CHM data is more accurate. In this study, the ResU-Net model with RGB-CHM combination provided the most accurate estimates of crown width (*R*^2^ = 0.9271, RMSE = 0.1282 m, rRMSE = 6.47%) and crown projection area (*R*^2^ = 0.9498, RMSE = 0.2675 m^2^, rRMSE = 9.39%). In conclusion, the combination of the deep learning model ResU-Net and UAV images containing elevation information has great potential for extracting crown information from *C. oleifera*. This method can obtain high-precision information on the tree crowns of *C. oleifera* trees at a low cost and with a high degree of efficiency, making it ideal for the precise management and rapid yield estimation of *C. oleifera* forests.

## Data availability statement

The raw data supporting the conclusions of this article will be made available by the authors, without undue reservation.

## Author contributions

YJ and DM: conceptualization. YJ: data curation and writing—original draft. YJ and XY: investigation and methodology. DM, YS and WW: resources and supervision. XY: validation. YJ and EY: writing—reviewing and editing. All authors contributed to the article and approved the submitted version.

## Funding

This research was funded by the National Natural Science Foundation of China (grant numbers: 31901311 and 32071682) and Science and Technology Innovation Plan Project of Hunan Provincial Forestry Department (grant number XLK202108-8).

## Conflict of interest

The authors declare that the research was conducted in the absence of any commercial or financial relationships that could be construed as a potential conflict of interest.

## Publisher’s note

All claims expressed in this article are solely those of the authors and do not necessarily represent those of their affiliated organizations, or those of the publisher, the editors and the reviewers. Any product that may be evaluated in this article, or claim that may be made by its manufacturer, is not guaranteed or endorsed by the publisher.

## Footnotes

^1^ https://www.dji.com/phan-tom-4-rtk
